# Prenatal substance exposure and child health: Understanding the role of environmental factors, genetics, and brain development

**DOI:** 10.1093/pnasnexus/pgae003

**Published:** 2024-01-30

**Authors:** Zixin Gu, Deanna M Barch, Qiang Luo

**Affiliations:** National Clinical Research Center for Aging and Medicine at Huashan Hospital, State Key Laboratory of Medical Neurobiology and MOE Frontiers Center for Brain Science, Institutes of Brain Science and Human Phenome Institute, Fudan University, Shanghai 200032, China; MOE Key Laboratory of Computational Neuroscience and Brain-Inspired Intelligence, Institute of Science and Technology for Brain-Inspired Intelligence, Fudan University, Shanghai 200433, China; Department of Psychological and Brain Sciences, Washington University in St Louis, St Louis, MO 63130, USA; Department of Psychiatry, Washington University in St Louis, St Louis, MO 63110, USA; Department of Radiology, Washington University in St Louis, St Louis, MO 63110, USA; MOE Key Laboratory of Computational Neuroscience and Brain-Inspired Intelligence, Institute of Science and Technology for Brain-Inspired Intelligence, Fudan University, Shanghai 200433, China; Shanghai Research Center of Acupuncture & Meridian, Shanghai 200433, China

**Keywords:** prenatal substance exposure, health in context, adolescence, brain development

## Abstract

Prenatal substance exposure (PSE) has been linked to adverse health outcomes, but its interactions with environmental and genetic factors remain unclear. Using data from the adolescent brain cognitive development cohort (*n* = 9,838; baseline age: 9.92 ± 0.62 years), we tested for the robust associations of PSE-caffeine/alcohol/tobacco/marijuana with children's health, cognition, and brain metrics after controlling for the environmental and genetic contexts. The environmental context involved birth, familial, and societal risk factors, while the genetic context included family histories and polygenic risk scores (PRSs) of mental disorders. In this sample, PSE-caffeine was observed in 59.8%, PSE-alcohol in 25.7%, PSE-tobacco in 13.2%, and PSE-marijuana in 5.6% of children. PSE-tobacco/marijuana was associated with higher environmental risks, PSE-alcohol was associated with lower familial risks, and all PSEs were associated with higher genetic risks. Controlling for these contexts reduced the number of significant health associations by 100, 91, 84, and 18% for PSE-tobacco/marijuana/caffeine/alcohol. Compared to the baseline, PSE-alcohol had the most health associations that were persistent over a 2-year period from preadolescence to adolescence, including associations with more sleep and mental health problems, improved cognitive functions, and larger brain volumes. These persistent associations with mental health problems and crystallized cognition were mediated by the surface areas of the frontal and the parietal cortices, respectively. Lower risk scores of the familial contexts attenuated associations between PSE-alcohol/marijuana and mental health problems. Higher PRS for substance use disorders enhanced late-onset associations of PSE-marijuana with externalizing problems. Results support the “health in context” concept, emphasizing modifiable factors mitigating adverse PSE effects.

**Significance Statement**

This study illuminates the health associations of prenatal substance exposure (PSE) in children from age 10 to 12, considering environmental and genetic contexts. Analysis of adolescent brain cognitive development study data uncovers robust associations between PSE and neurobehavioral problems, cognitive abilities, and brain metrics. Notably, controlling for environmental and genetic contexts diminishes a substantial number of associations, highlighting their confounding roles. PSE-alcohol stands out as the primary substance exhibiting persistent associations, while PSE-marijuana reveals late-onset associations. Surface areas of brain regions in frontal and parietal cortices, along with potentially modifiable familial context were identified as potential contributors along the pathways of PSE effects.

## Introduction

Prenatal substance exposure (PSE) has been associated with risks for mental health disorders (1), developmental disabilities (2), and mortality (3) in both children and adolescents. In the United States, 9.8% of pregnant women who reported alcohol consumption also disclosed simultaneous smoking (28.1%) or marijuana use (20.6%) (4). However, the pathways through which PSEs impact child and adolescent health remain elusive. This is mainly due to the co-occurrence of PSEs with diverse substances and their intricate interactions with various environmental and genetic factors (5). Understanding the biological pathways in the context of these complex interactions could potentially lead to the development of intervention strategies targeting modifiable elements.

Some reported effects of PSE on child and adolescent health might be confounded with environmental and genetic risk factors that co-occur with PSEs. For example, adjusting for socioeconomic status and parental psychopathology diminished the association between PSE-tobacco and behavioral disorders (5). To date, no study has systematically examined the impact of co-occurring environmental and genetic context effects on the associations of PSE with child-health outcomes. The data being acquired as part of the adolescent brain cognitive development (ABCD) study enables us to identify the robust health associations of PSE by more comprehensively examining the potential confounding effects of these contexts.

Previous studies using the ABCD cohort have reported various child-health associations with PSE-caffeine (6), PSE-alcohol (7), PSE-tobacco (8), and PSE-marijuana (9). However, these studies considered different sets of context factors, making it challenging to compare the health association patterns among PSE to different substances. Another limitation of these previous findings is the use of only the baseline data when children were 9 and 10 years old. Examination of how the health associations of PSE evolve across development is essential for interventions that target persistent or later occurring outcomes over transient ones.

It is also important to understand the pathways by which PSEs may influence child-health outcomes, including potential neural mechanisms. Recent studies using the ABCD cohort revealed that structural brain indices were associated with negative psychological and behavioral outcomes and acted as a partial mediator of associations of PSE-alcohol/caffeine with externalizing problems in cross-sectional models (6, 7). However, to better understand potential causal relationships, examining mediation using longitudinal data is important to understand the mechanisms contributing to persistent/late-onset health associations of PSE in children.

In addition to understanding what PSE to child-health outcomes might or might not reflect co-occurring environmental or genetic risk factors, it is also important to explore the interactions of environmental and genetic contexts with PSE in relation to various health outcomes in children. For example, it has long been suggested in the literature that the interactions between genetic risks for alcohol-use disorders and prenatal alcohol/tobacco exposure (10) are associated with adolescent health, such as substance misuse (11). Importantly, environmental factors, such as poverty, parenting stress, and family functioning can either attenuate (12) or strengthen (13) the associations between prenatal substance exposures and health problems in children, and some of these factors may be modifiable. Therefore, delineating the moderating roles of these context factors in the persistent/late-onset health associations of PSE is crucial to identifying potential pathways to attenuate the impact of PSEs on child-health outcomes.

**Fig. 1.**
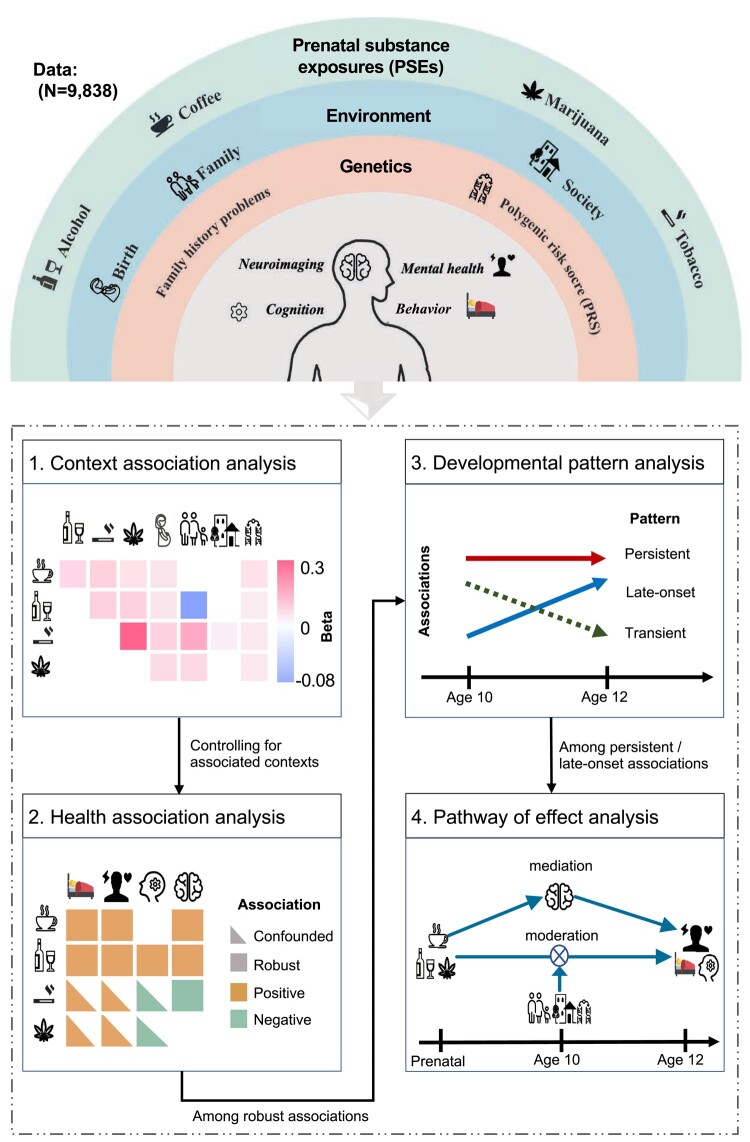
Analysis scheme. This study examines the health associations of four common PSEs in the contexts of early-life environment, family and society environment, and individual genetic factors. The health outcomes are behavioral, psychopathological, cognitive, and neuroimaging measures in preadolescents. Analysis 1: context association analysis, identifying the context factors that are associated with prenatal substance exposures. Analysis 2: health association analysis, identifying robust associations of PSEs with health outcomes at the baseline after controlling for the potential confounding effects of the associated context factors. Analysis 3: developmental pattern analysis, comparing the baseline and the follow-up health associations of PSEs to identify persistent and late-onset associations. Analysis 4: pathway of effect analysis: detecting brain mediation and context moderation effects on those persistent/late-onset associations.

This study aims to understand the child-health associations of the four most common prenatal substance exposures—caffeine, alcohol, tobacco, and marijuana—in the context of brain development, environment, and genetics. PSE was obtained by parental retrospective reports regarding maternal substance use before and after pregnancy knowledge in the ABCD study. We generated a binary variable to indicate the presence or absence of substance use, estimated the total consumption of each substance during pregnancy, and defined patterns of substance use (e.g. light or heavier use, as well as reducing, stable, or increasing use). Using the longitudinal data of the ABCD cohort, we first systematically examined the associations among PSE to different substances and their relationships with child-health outcomes and with potential environmental and genetic context risk factors. Second, we delineated the robust health associations of PSE at baseline by controlling genetic and environmental factors that may co-occur with PSEs. Third, we analyzed the follow-up data to distinguish between transient and persistent/late-onset health associations of PSE. Finally, we performed pathway analyses to test the brain mediation and environmental or genetic moderation effects of persistent/late-onset health associations of PSE (Fig. 1).

## Results

### PSEs were highly associated with each other

**Fig. 2.**
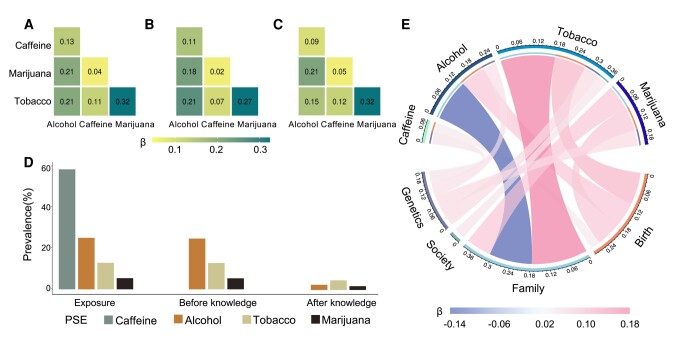
Context associations. A) Associations among PSEs. These numbers reflect standardized *β* coefficients. B) Associations among PSEs before knowledge of pregnancy. C) Associations among PSEs after knowledge of pregnancy. The values again represent standardized *β* coefficients. D) Prevalence of substance use during pregnancy. E) Associations of PSEs with environmental and genetic risk-factor context scores in preadolescent children. The values again represent standardized *β* coefficients. Birth = birth context score; family = family context score; society = society context score; PGS = genetic context score.

Demographic characteristics are provided in Table 1 and means and standard deviations for behavioral, mental health, and cognitive measures in youth are provided in Table S2 in the supplement. Of the 9,838 children (47.9% female) included in this study, 2,524 (25.7%) had PSE to alcohol, 5,880 (59.8%) to caffeine, 1,300 (13.2%) to tobacco, and 547 (5.6%) to cannabis (Fig. 2D). PSE-marijuana was associated with PSE-tobacco, and PSE-alcohol was associated with PSE-tobacco, marijuana, and caffeine (Table S3 and Fig. 2A). These findings were similar when we separately examined PSE before and after learning of pregnancy (Fig. 2B and C). PSE-alcohol was significantly lower post than prepregnancy (Fig. 2D).

**Table 1.** Demographic characteristics of children and adolescents (*n* = 9,838).

	No exposure	Caffeine		Alcohol		Tobacco		Marijuana		Polysubstance	
	(*n* = 3,042)	(*n* = 5,880)		(*n* = 2,524)		(*n* = 1,300)		(*n* = 547)		(*n* = 2,587)	
	*n* (%)	*n* (%)	*P*	*n* (%)	*P*	*n* (%)	*P*	*n* (%)	*P*	*n* (%)	*P*
Sex			0.312		0.086		0.104		0.104		0.159
Female	1,428 (47%)	2,828 (48%)		1,244 (49%)		278 (51%)		278 (51%)		1,264 (49%)	
Male	1,614 (53%)	3,052 (52%)		1,280 (51%)		269 (49%)		269 (49%)		1,323 (51%)	
Race/ethnicity			**<0.001**		**<0.001**		**<0**.**001**		**<0**.**001**		**<0**.**001**
Asian	89 (3%)	84 (1%)		30 (1.2%)		4 (1.0%)		4 (1.0%)		22 (1%)	
Black	573 (19%)	612 (10%)		214 (9%)		167 (31%)		167 (31%)		309 (12%)	
Hispanic	761 (25%)	1,042 (18%)		397 (16%)		106 (19%)		106 (19%)		395 (15%)	
White	1,334 (44%)	3,509 (59%)		1,640 (65%)		211 (39%)		211 (39%)		1,555 (60%)	
Other	285 (9%)	633 (11%)		243 (10%)		59 (11%)		59 (11%)		306 (12%)	

	Mean (SD)	Mean (SD)	*P*	Mean (SD)	*P*	Mean (SD)	*P*	Mean (SD)	*P*	Mean (SD)	*P*
Age at baseline (years)	9.92 (0.62)	9.92 (0.62)	0.542	9.91 (0.63)	0.759	9.88 (0.61)	0.147	9.88 (0.61)	0.147	9.93 (0.63)	0.328
Birth context score	−0.06 (0.57)	0.02 (0.61)	**<0.001**	0.06 (0.64)	**<0.001**	0.50 (0.72)	**<0.001**	0.50 (0.72)	**<0.001**	0.16 (0.65)	**<0.001**
Family context score	0.01 (0.70)	−0.01 (0.70)	0.086	−0.12 (0.68)	**<0.001**	0.47 (0.73)	**<0.001**	0.47 (0.73)	**<0.001**	0.07 (0.75)	**0.006**
Society context score	0.05 (0.75)	−0.04 (0.71)	**<0.001**	−0.02 (0.71)	**0.02**	0.28 (0.69)	**<0.001**	0.28 (0.69)	**<0.001**	0.04 (0.70)	**0.372**
Genetic context score	−0.44 (1.65)	0.21 (1.78)	**<0.001**	0.44 (1.75)	**<0.001**	0.87 (2.03)	**<0.001**	0.87 (2.03)	**<0.001**	0.56 (1.84)	**<0.001**
PRS	0.59 (0.11)	0.47 (0.12)	**<0.001**	0.59 (0.11)	**<0.001**	0.58 (0.11)	0.076	0.58 (0.11)	0.076	0.58 (0.11)	0.076
ADHD	0.46 (0.12)	0.47 (0.12)	0.172	0.47 (0.12)	0.759	0.49 (0.12)	**0**.**002**	0.49 (0.12)	**0**.**002**	0.47 (0.12)	0.068
Schizophrenia	0.61 (0.14)	0.61 (0.13)	0.708	0.62 (0.13)	0.765	0.63 (0.13)	0.39	0.63 (0.13)	0.39	0.62 (0.13)	0.842
Major Depressive Disorder	0.42 (0.12)	0.41 (0.11)	**<0.001**	0.40 (0.11)	**<0.001**	0.43 (0.12)	0.08	0.43 (0.12)	0.41	0.41 (0.11)	**0**.**002**
Alcohol dependence	0.49 (0.14)	0.49 (0.13)	0.829	0.48 (0.14)	0.938	0.50 (0.13)	**0**.**036**	0.51 (0.14)	**0**.**037**	0.49 (0.13)	0.872
Cannabis use disorder	0.59 (0.11)	0.59 (0.11)	**0.024**	0.59 (0.11)	0.059	0.58 (0.11)	0.263	0.59 (0.10)	0.34	0.59 (0.11)	0.112
Educational attainment	−0.44 (0.12)	−0.44 (0.11)	0.069	−0.44 (0.11)	0.938	−0.39 (0.11)	**<0.001**	−0.40 (0.12)	**<0.001**	−0.43 (0.12)	**0**.**002**

Significant *P*-values (*P* < 0.05) were bolded.

### PSEs were differentially associated with environmental and genetic contexts

We found that PSEs were associated with higher birth context risk-factor scores and higher genetic context risk-factor score, with the later primarily attributable to a greater family history of psychiatric disorders (Table S1). PSE-alcohol was associated with a lower family risk-factor context score, whereas PSE-tobacco/marijuana was associated with a higher family context score. Only PSE-tobacco was associated with a higher society risk-factor context score (Fig. 2E and Table S4). The PSE associations with the society risk-factor context score were mainly driven by lead risk level, neighborhood safety, and child opportunity index (Table S1).

### Robust health association of PSEs at baseline

**Fig. 3.**
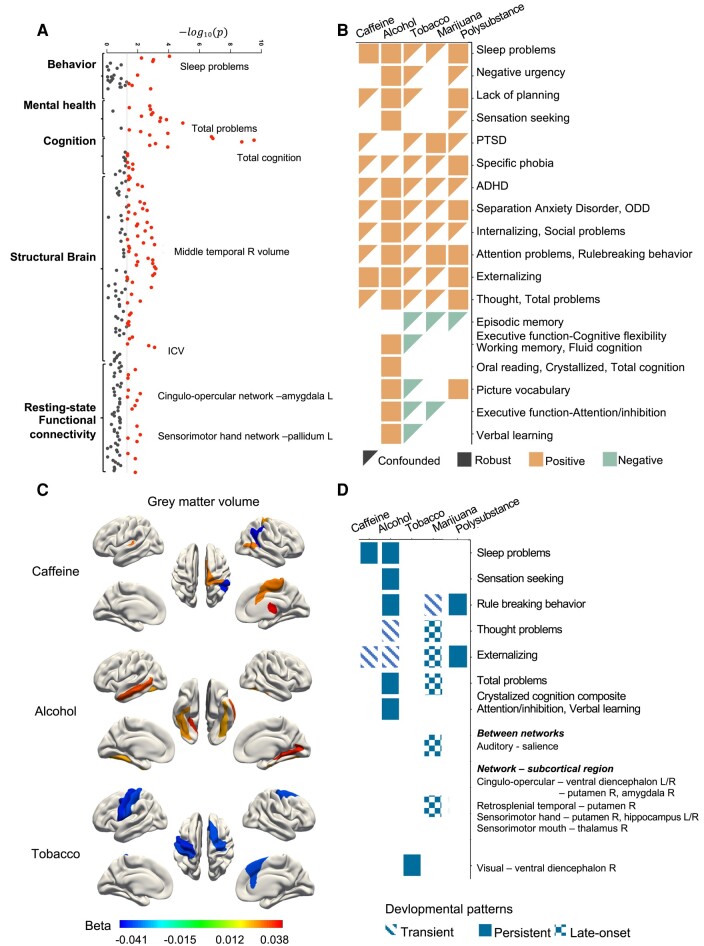
Associations of PSEs with behavioral, mental, cognitive, and neuroimaging outcomes. A) Manhattan plot of the largest –log10(*P*-values) of the health associations among four PSE and polysubstance use at the baseline. Significant *P*-values (*P* < 0.05 after FDR correction) were highlighted. B) Significant health associations of each PSE after FDR correction in preadolescents at the baseline. Shapes represent associations that remained significant after controlling for context variables (squares) or were confounded by context variables (triangles). C) Significant regional gray matter volumes associated with the PSE-alcohol/caffeine/tobacco (*P* < 0.05 after FDR correction). D) Developmental patterns of PSEs’ associations from preadolescence to adolescence.

After controlling for both environmental and genetic context risk-factor scores, we found that many PSEs associations with child outcome measures at baseline remained significant (*P*_FDR_ < 0.05; Fig. 3A and B, and Tables S5 and S6). PSE-alcohol had the most associations, with relation to more sleep problems, higher trait negative urgency and sensation seeking, lack of planning, and more emotional and behavioral problems (*β*: 0.03–0.06), but better cognitive functions (*β*: 0.03–0.09). PSE-caffeine was associated with more sleep problems and more externalizing problems (*β*: 0.03–0.05). All health associations with PSE-tobacco became nonsignificant when controlling for the environmental and genetic contexts. The prenatal polysubstance exposure was associated with more sleep problems, emotional and behavioral problems (*β*: 0.04–0.07), and better cognitive functions (*β*: 0.04–0.06). When we separately examined the PSE before and after learning of pregnancy, adolescents with PSE-marijuana after learning of pregnancy had more externalizing problems than those with PSE-marijuana before learning of pregnancy (*β*: 0.05; Tables S11 and S12).

### Persistent/late-onset health association of PSEs at 2-year follow-up

In the analyses of year 2 outcomes, there were a number of associations that were no-longer significant and significantly reduced compared to baseline (Tables S5 and S6). We observed persistent associations of PSE-caffeine/alcohol with more sleep problems, PSE-alcohol with better-crystalized intelligence of similar effect sizes as those at baseline, and prenatal polysubstance exposure with increased attention and externalizing problems (Fig. 3D and Tables S5 and S6). The effect size of PSE-alcohol on total problems reduced significantly (*β*_2-year_ − *β*_baseline_ = −0.035, 95% CI: [−0.063, −0.008]), whereas the effect size of PSE-marijuana significantly increased (*β*_2-year_ − *β*_baseline_ = 0.034, 95% CI: [0.001, 0.067], Fig. 3D and Tables S5 and S6).

### Linear dose–response health associations of PSEs at 2-year follow-up

At the 2-year follow-up, linear dose–response associations were observed, indicating that increased PSE-caffeine consumption was associated with more sleep problems. Increased PSE-alcohol consumption was associated with higher sensation seeking, more sleep problems, and total problems. Similarly, more frequent PSE-marijuana was associated with increased thought, externalizing, and total problems (Fig. S2 and Table S6). Three patterns of caffeine intake, five patterns of alcohol use, and five patterns of smoking during pregnancy were identified. These patterns encompassed a spectrum from light to heavier use and from reducing and stable to increasing use (Fig. S1). As compared with the controls without reporting PSE, the daily/weekly PSE-caffeine was associated with more sleep problems, the stable light PSE-alcohol was associated with more sleep problems and total mental problems, and light/heavier reducers with PSE-alcohol showed better-crystallized cognition (Table S6).

### Robust and persistent structural brain associations of PSEs

After controlling for the context scores, we identified robust associations of PSE-caffeine/alcohol/tobacco with a number of brain structure metrics after false discovery rate (FDR) correction at baseline (*P*_FDR_ < 0.05; Fig. 3A and C and Tables S7 and S8). At year 2, PSE-alcohol had persistent associations with larger intracranial volume, regional cortical volumes in the bilateral middle temporal gyri, global, and regional cortical surface areas in the inferior parietal, fusiform, right lateral orbital frontal, frontal pole, precuneus, superior temporal, transverse temporal, left inferior temporal, middle temporal, and lateral occipital gyri. PSE-tobacco was persistently associated with smaller global and regional cortical volumes in the precentral and left postcentral gyri and regional cortical surface areas in the left precentral, postcentral and right superior frontal, entorhinal, and posterior cingulate gyri. PSE-caffeine was persistently associated with larger right postcentral, precuneus, supramarginal gyri, smaller surface area in the right pericalcarine cortex, larger surface areas in the right superior parietal and supramarginal gyri, and thicker left isthmus-cingulate cortex (all *P*_FDR_ < 0.05; Fig. 3C and Tables S7 and S8). Structural neural indices did not show significant associations with prenatal polysubstance exposure at baseline (Tables S7 and S8). Total substance consumption had similar linear associations with structural neuroimaging measures (Table S8).

### Late-onset/persistent functional brain associations of PSEs

Controlling for the context scores, we found functional brain associations of PSEs after FDR correction (*P*_FDR_ < 0.05) at baseline (Table S9) and 2-year follow-up (Table S10). We observed nine late-onset functional connectivity associations of PSE-marijuana, mainly from cingulo-opercular and sensorimotor networks to subcortical regions (Fig. 3D and Table S10). There were no persistent associations of PSE-alcohol/tobacco, but we observed 14 transient associations of PSE-tobacco, mainly from connections in the sensorimotor network, cingulo-opercular network, and visual network (Table S9), as well as two transient associations of PSE-alcohol (Table S9).

### Brain measures at baseline mediated the persistent/late-onset health associations of PSEs

**Fig. 4.**
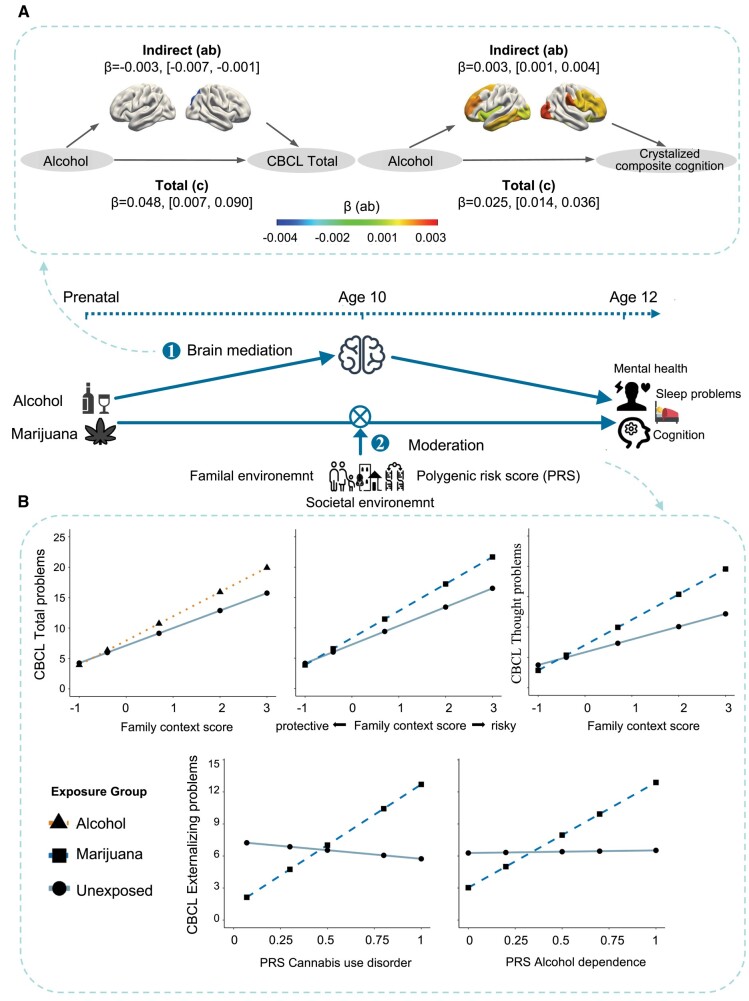
Pathways of effects for the persist/late-onset health associations of prenatal substance exposures. A) Left: regional cortical surface areas at the baseline showing significant mediation effects on the associations of PSE-alcohol with CBCL total problems at the follow-up; right: regional cortical surface areas at the baseline showing significant mediation effects on the associations of PSE-alcohol with crystalized composite cognition at the follow-up. Brain regions were shown according to their indirect effects measured by *β*-values. B) Lower family risk context scores attenuated the associations of PSE-alcohol/marijuana with total/thought problems; higher PRS for cannabis use disorders/alcohol dependence strengthened the associations of PSE-marijuana with externalizing problems.

We tested longitudinal mediation effects of baseline structural brain measures on the 2-year follow-up health associations of PSEs. After FDR correction, we found that the global surface area (ratio = −7.20%, 95% CI: [−0.007, −0.001]) and the surface area of the right inferior parietal gyrus (ratio = −7.09%, 95% CI: [−0.008, −0.001]) mediated the associations of PSE-alcohol with total problems and some of the identified structural measures mediated the associations of PSE-alcohol with crystalized composite cognition (i.e. the intracranial volume, global, and cortical surface areas in the rostral middle-frontal, superior frontal, fusiform, middle temporal and insula; all bootstrapped *P* < 0.05; ratio: 3.60–10.53%; Fig. 4A and Table S16).

### Context scores at baseline moderated the persistent/late-onset health associations of PSEs

After FDR correction, we identified significant interactions between family context scores and PSEs for a number of child-health outcomes. Lower family context scores at baseline attenuated the associations of PSE-alcohol with total mental health problems from the child behavior checklist (CBCL; VR^2^ = 0.47; bootstrapped *P* = 0.001) and the associations of PSE-marijuana with total and thought problems from the CBCL (VR^2^ = 0.45/0.62; all bootstrapped *P* < 0.05). We also found interactions of genetic risk-factor scores and PSE for several outcomes. Higher polygenic risk scores (PRS) for cannabis use disorders or alcohol dependence strengthen the associations of PSE-marijuana with more externalizing problems (VR^2^ = 0.04 or 0.08; all bootstrapped *P* < 0.05; Fig. 4B and Table S17).

## Discussion

This research is among the first to systematically compare the developmental patterns of the health associations of prenatal exposures to four common substances in the context of both environmental and genetic risk-factor contexts. Notably, our findings revealed that associations of PSE-tobacco became statistically insignificant after considering environmental and genetic factors. However, a number of associations with PSE-caffeine/alcohol/marijuana were robust and remained when controlling for environmental and genetic context. Further, our longitudinal analyses uncovered distinct developmental patterns, including persistent, transient, and late-onset associations. Our mediation pathway analyses revealed that larger surface areas of the frontal and parietal cortices at 10 years old mediated the persistent associations of PSE-alcohol with more mental health problems and better-crystallized intelligence. Notably, lower risk scores for the family context and lower PRS for substance use disorders significantly reduced the persistent/late-onset associations of PSE-alcohol/marijuana with more mental health problems, respectively.

Our comprehensive examination of the genetic and environmental contexts revealed both confounded and robust health associations of the four common prenatal substance exposures. Given the complex nature of health, there has been increasing attention focused on the concept of “health in context” (14). However, previous studies often considered different sets of genetic and environmental factors in their studies of prenatal exposures to different substances (6–9), which has made it difficult to compare these confounding effects among different substances. In one unified comprehensive study, our findings revealed that both birth and family context scores had the most confounding effects on the health associations of PSE-tobacco followed by PSE-marijuana, while they had the fewest confounding effects for PSE-alcohol/coffee. Further, various genetic contexts also have confounding effects on the associations of all four types of PSEs with child outcomes. Before controlling for environmental and genetic contexts, we found 48 associations of PSE-tobacco. Strikingly, none of these associations remained significant after considering the context scores. This finding is supported by the previous study with a sibling design demonstrating that the substantial familial or genetic confounded the association of attention deficit hyperactivity disorder (ADHD) with PSE-tobacco in offspring (15). For PSE-marijuana, the genetic contexts confounded 33 health associations, which are supported by the literature reporting a shared genetic liability between cannabis use disorder and psychiatric disorders such as ADHD and schizophrenia (16). These findings highlighted the importance of filtering out potentially confounded associations by examining the context confounding to focus on the robust health associations of PSEs.

After systematically controlling for birth, family, society, and genetic contexts, the current study confirmed robust health associations of PSE-alcohol with both more sleep problems and mental health problems (e.g. internalizing, externalizing, total, thought, and attention). However, intriguingly, PSE-alcohol was associated with better cognitive test scores at the age of 10 years, which was also reported in a previous study of PSE-alcohol (7). We also found some robust health associations of PSE-caffeine with more sleep problems and externalizing problems (6). However, for PSE-marijuana, the only robust association was with attention problems at the age of 10 years old, and most of the other health associations reported in the literature (9) were confounded by genetic and environmental contexts. However, we did find that PSE-marijuana after maternal knowledge of pregnancy, indicating more sustained and severe marijuana use, was associated with more externalizing problems and thought problems even after controlling for context scores.

Our findings of the developmental patterns of the robust health associations of PSEs identified a few persistent associations. Most studies examined the health associations of PSEs at specific ages, and few studies studied the developmental patterns of these associations. One study (*n* = 2,900) found a lasting but gradually reducing effect of PSE-alcohol on mental health problems in children between 2 and 14 years old (17). The current finding provided new evidence some effects remained at year 2. A previous study has reported that PSE-caffeine has a persistent association with sleep problems in children from 6 months to 8 years old (18). Our finding extended this persistent association to 10 and 12 years old. Sleep problems have been associated with both neurocognitive development (19) and psychotic experiences in children (20), inattention in adolescents (21), and depression in adults (22). This persistent association of PSE-caffeine with sleep problems in children calls for further studies with more detailed information on the frequency and amount of use in mothers of this common substance and to better understand its impact on offspring health. It has long been hypothesized in literature that the effects of PSE-marijuana on offspring health may manifest in a gradually increasing way as the children grow up (23). Our finding of a late-onset association between PSE-marijuana and total mental health problems (*β* significantly increased from 0.02 at 9–10 years old to 0.05 at 11–12 years old) provided new empirical evidence for this hypothesis.

Notably, in the literature, the PSE-alcohol has been associated with smaller volumes of some brain structures (24). After comprehensively controlling for the environmental and genetic contexts that are relevant to offspring health, our findings confirmed brain structural associations of PSE-alcohol at the age of 10 years old. However, different from the previous studies, we found that PSE-alcohol was associated with larger gray matter volumes rather than smaller volumes. This may be partially due to the fact that in the previous studies, the prenatal alcohol exposures were examined in populations with a diagnosis of fetal alcohol spectrum disorder (25) or with very heavy prenatal exposure to alcohol (26) much greater than the level of population-based PSE-alcohol in this study. Notably, we found persistent associations of both more total mental health problems and better-crystalized composite cognition with PSE-alcohol. It has been hypothesized in the literature that these findings might reflect a compensatory response of some brain regions to counteract the impact of maternal alcohol use on other brain regions with disrupted function (7). This hypothesis may be supported by our longitudinal mediation finding of the right inferior parietal gyrus, the greater surface area of which reduced the association between PSE-alcohol and mental health problems. However, this study could not identify any structural neuroimaging measures acting as a neural mediator to facilitate the association between PSE-alcohol and more mental health problems, which remains an open question. Nevertheless, facilitating the growth of these brain structures, for example the supportive touching of infants to promote the development of the frontal and parietal brain regions (27), might provide new opportunities for neuroimaging-informed precision intervention for children with PSE-alcohol.

Our findings of environmental moderation effects on the health associations of PSEs emphasized the importance of focusing on these potentially modifiable environmental factors. Family environment has long been associated with children's health outcomes, including family conflict (28), parenting behavior (29), and socioeconomic status (30), etc. However, it remains unclear to what extent the family environment could moderate the health associations of PSEs. Here, using the environmental context score related to risk factors within the family assessed at the age of 10 years old, we found that when the family risk score was below its population mean, it significantly attenuated the PSE-alcohol/marijuana associations with mental health problems at age 12 years old (VR^2^ > 45%). These findings provided new empirical evidence supporting the view of social determinants of health (31) and indicate that the impact of PSE may differ based on both environmental and genetic context.

This study has some limitations. Responses to substance use during pregnancy are based on self-reports, which include answers regarding the timeline that may not be accurate. The use of substances during pregnancy may be underreported in self-reported measures, since many women may choose not to disclose prenatal substance use (32). This might also be the reason for the low prevalence (5.6%) of marijuana use in the current sample as this prevalence increased from 3.4 to 7.0% between 2002 and 2017 in the United States (33). For health guidelines, the timing-effect is also an important issue for PSE and may have specific negative consequences (34). However, because the ABCD did not ask about the specific trimester of use, we could not include the timing of exposures in terms of trimester in this study.

In summary, this study revealed not only the persistent health associations of prenatal exposure to alcohol and caffeine and the late-onset health association of prenatal exposure to marijuana but also the neurodevelopment mediation and the family moderation effects on these associations. These findings emphasized the important roles of genetics, environment, and brain in the health association studies of PSE.

## Materials and methods

### Participants

Data from the ABCD study dataset annual version 4.0.1 was used (baseline data collection from 2016 September 1 to 2018 October 15; 2-year follow-up data collection from 2018 July 30 to 2020 January 15). This is a large longitudinal study that invited 11,877 children aged 9–11 years from different geographic, demographic backgrounds, and socioeconomic status across 22 sites in the United States beginning in 2018 (35). All parents/guardians and children provided their written-informed consent and written assent. The study obtained approval from the Central Institutional Review Board at the University of California, San Diego. A total of 9,838 participants were included in the analyses due to the exclusion of incomplete data and brain scans that failed quality controls conducted by the ABCD study (Fig. S1).

### Prenatal substance exposures

Prenatal substance exposures were measured using the developmental history questionnaire, in which mothers retrospectively reported any alcohol, caffeine, marijuana, and tobacco use during pregnancy, the quantity of caffeine consumption per day/week/month and the frequency of caffeine consumption, the maximum number of drinks consumed on a single occasion and the average number of drinks consumed per week, and the frequency of smoking and marijuana use per day during pregnancy before and after knowledge of the pregnancy (36). The children of those mothers who reported “yes” either before or after knowledge of pregnancy were grouped as having prenatal exposure to the substance. Exposure after knowledge of pregnancy refers to those with or without exposure to the substance before knowledge. We further derived the polysubstance exposure and coded it as “yes” if more than one substance was used during pregnancy. The total consumption of substances during pregnancy was estimated for each substance, such as the total cups of caffeinated drinks, the total number of alcoholic drinks, and the total instances of marijuana or tobacco use. Applying the cutoffs as recommended by the literature to our data (7, 37), the substance use can be classified as light or heavier use according to the total consumption. Meanwhile, substance use can also be classified as reducing, stable, and increasing use by comparing the total consumption before and after the knowledge of pregnancy. Bringing these two classifications together, we defined five patterns of substance use for alcohol and tobacco. The patterns of caffeine use were daily, weekly, and monthly. The information on caffeine use before and after the knowledge of pregnancy was not collected by the ABCD study. As the daily use of marijuana has already been considered heavy use, we did not further divide it into different categories. Additional details and relevant questions from the ABCD protocol were provided in the supplement.

### Health outcomes

#### Behavior measures

The sleep disturbance scale for children was used to examine total sleep problems (36). Impulsivity was assessed using the urgency, premeditation, perseverance, sensation seeking, positive urgency, and impulsive behavior (UPPS-P) scale for children (36). Inhibition and reward-seeking were examined using the Behavioural Inhibition System and Behavioural Activation System (BIS/BAS) scales (36). The prosocial behavior of children and adolescents was assessed using the prosocial behavior scale (36).

#### Mental health measures

Dimensional psychopathology was examined in children using the eight empirically based syndrome scales of the parent-reported CBCL (36), assessing anxious/depressed, withdrawn/depressed, somatic problems, rule-breaking, aggressive behavior, internalizing, externalizing, and total problems. Psychiatric disorders (i.e. diagnoses and/or symptoms, past, and/or present) were determined according to the Diagnostic and Statistical Manual of Mental Disorders, 5th ed. (DSM-5) criteria using parent-reported responses on the schedule for affective disorders and schizophrenia in school-aged children (K-SADS) (36).

#### Cognitive measures

All individual tests from the NIH toolbox battery were utilized, including the dimensional change card sort, flanker inhibitory control and attention, list sorting working memory, oral reading recognition, pattern comparison processing speed, picture sequence memory, and picture vocabulary tests, as well as total, crystallized, and fluid composites (38). The Rey auditory verbal learning test was used to measure learning and memory recall (39). Risk-taking was examined using the single-item cash choice task (40) while the little man task assessed visuospatial processing, flexibility, and attention (41).

#### Structural brain measures

Neuroimaging metrics were evaluated on 3T scanners across the ABCD study sites. Participants completed MRI scans of 3D T_1_-weighted images with correction (35). Cortical surface reconstruction was processed by FreeSurfer, version 5.3.0. In the present study, we focused on cortical volumes, surface areas, and thicknesses of 68 brain regions from the Desikan–Killany atlas for imaging analyses.

#### Resting-state functional correlations

The Gordon functional atlas was utilized to categorize cortical surface regions into 12 large networks, including auditory, cingulo-opercular, cingulo-parietal, default-mode, dorsal-attention, fronto-parietal, retrosplenial-temporal, salience, sensorimotor-hand, sensorimotor-mouth, ventral-attention, and visual networks (42). To determine the strength of resting-state functional connectivity (rsFC), the Fisher *r*-to-*z* transformed indices of the average correlation values were computed between pairs of regions within each large network (*n* = 12), between these 12 networks (*n* = 66), and between the large networks and 19 subcortical regions (*n* = 228).

### Environmental and genetic context scores

The environmental context encompasses factors related to birth, family, and society (14). A birth risk-factor score was calculated by considering factors such as planned birth, other substance usage (e.g. cocaine, heroin), vitamin intake during pregnancy, and duration of breastfeeding. In sensitivity analyses, we further included more early-life risk factors such as whether the child was born prematurely (yes, no, unknown), experienced birth complications (yes, no, unknown), or if any obstetric complications occurred during pregnancy (yes, no, unknown) in the birth context. The family risk-factor context score incorporated parental ages at birth, parental partnership, parental education level, and family income. The society risk-factor context score included neighborhood factors (e.g. noise and safety), urbanity, lead risk, air pollution, drug accessibility, and child opportunity scores. Genetic context score was established based on genetic covariates, including a family history of psychiatric disorders (e.g. depression, mania) and polygenic risk scores (43) (ADHD, schizophrenia, major depressive disorder, alcohol dependence, cannabis use disorder, and educational attainment). These variables, which were grouped into risky and protective categories, were normalized between 0 and 1 by min–max normalization to make these variable values comparable and additive. These normalized variables were then added together by their respective contexts, i.e. the birth, family, society, and genetic contexts. To ensure that higher scores indicated higher risk in each context, the normalized variables in the respective category were subtracted from instead of added to these scores. Below, we illustrate the context score derivation form using the birth context score as an example, and further details are described in the supplement:


$$\begin{array}{rl} \mathrm{Birth}\;\mathrm{context}\;\mathrm{score} & =\;{\mathrm{PSE}}_{\mathrm{cocaine}/\mathrm{crack}}+{\mathrm{PSE}}_{\mathrm{heroin}/\mathrm{morphine}}\\ & \;\;+{\mathrm{PSE}}_{\mathrm{oxycontin}}-{\mathrm{PSE}}_{\mathrm{vitamin}}\\ & \;\;+{\mathrm{Complications}}_{\mathrm{obstetric}}+{\mathrm{Complications}}_{\mathrm{birth}}\\ & \;\;+\mathrm{Prematurity}-\mathrm{Planned}\;\mathrm{pregnancy}\\ & \;\;-{\mathrm{Breastfeeding}}_{\mathrm{months}}. \end{array}$$

### Statistical analysis

Analyses included the 9,838 preadolescents who had complete data for the variables of interest and who had high-quality imaging data (Fig. S1). We tested the associations between PSEs and child-health outcomes at baseline and 2-year follow-up using linear mixed-effect models. Fixed-effect covariates included the essential covariates (i.e. sex at birth, race/ethnicity, and age at assessment). Random effects included nesting children within families to account for sibling effects and study sites. Intracranial volume was added as a fixed-effect covariate for volumetric analyses. Models for rsFC were additionally adjusted for head motion and image quality. We conducted sensitivity analyses to examine whether these associations differed between before and after the maternal knowledge of pregnancy using three groups: no exposure, exposure before knowledge of pregnancy only, and exposure after knowledge of pregnancy.

#### Test for confounding effects to identify robust health associations of PSEs

To test the potential confounding effects of co-occurring environmental and genetic contexts on these significant health associations of PSEs, we examined all four PSEs together and further adjusted for the birth, family, society, and genetic context scores as fixed-effect covariates in the mixed-effect models. We considered an association of PSE to child-health outcomes *robust* if it remains significant after controlling for co-occurring environmental and genetic risk-factors.

#### Examining developmental patterns to determine persistent/late-onset health associations of PSEs

Among the robust associations, we further examined their developmental patterns by comparing associations between the baseline and the 2-year follow-up. We obtained the 95% confidence interval of the difference between the effect sizes of the baseline association and the follow-up association by 10,000 bootstraps. If a robust association at the baseline remained significant at the follow-up, we considered it a *persistent association*. If a robust association was significantly reduced at the follow-up as compared to the baseline and was no longer significant at year 2, we considered it a *transient association*. If an association increased significantly from the baseline to the follow-up and was only significant at year 2, we considered it a *late-onset association*. We further investigated how persistent/late-onset health associations of PSE vary based on dosage and substance use patterns using linear mixed-effect models.

#### Testing mediation and moderation effects of the context scores on the persistent/late-onset health associations of PSEs

Focusing on the robust and persistent/late-onset health associations of PSEs, we tested whether any of the brain-metrics mediated associations of PSEs to child-health outcomes using the “BruceR” package. To remove the family dependency from the data, we randomly selected one child from each family for a new sample (*n* = 8,262). Covariates in the model were age, sex, race, and data collection site. We tested the significance of the interaction terms between the genetic or environmental context scores and PSEs to examine the moderation effects on the health associations of PSEs using the linear mixed-effect models described above.

In all analyses, false discovery rates were used to adjust for multiple comparisons with adjusted *P*-values reported (44). The linear mixed-effects model, mediation, and moderation analyses were performed using the R version 4.2.1(the “lmerTest” and “BruceR” packages).

## Supplementary Material

pgae003_Supplementary_Data

## Data Availability

Data are publicly released on an annual basis through the National Institute of Mental Health (NIMH) data archive (NDA, https://nda.nih.gov/abcd). The ABCD study data are openly available to qualified researchers for free. Access can be requested at https://nda.nih.gov/abcd/request-access. An NDA study has been created for the data used in this report under the doi: 10.15154/1528317. Code for the replication of analyses conducted in the manuscript can be retrieved at https://github.com/gzixin/Prenatal-substance-exposure-child-and-adolescent-health.
