# Supplementary material for: Prenatal substance exposure and child health: Understanding the role of environmental factors, genetics, and brain development
Source: PNAS Nexus. 2024 Jan 30;3(1):pgae003. doi: 10.1093/pnasnexus/pgae003 (PMC10826906; doi:10.1093/pnasnexus/pgae003)
Supplement: pgae003_Supplementary_Data [file pgae003_supplementary_data.zip › PNASNEXUS-PNASNEXUS-2023-00807R-s01.docx]

Supplemental information

[Method S1: Prenatal substance exposures 2](#_Toc152933913)

[**ABCD Baseline Substance Use Questions During Pregnancy** 2](#_Toc152933914)

[**Prenatal polysubstance exposure** 2](#_Toc152933915)

[**Total prenatal substance consumption** 2](#_Toc152933916)

[**Prenatal substance use patterns classification** 3](#_Toc152933917)

[Method S2: Health outcomes 4](#_Toc152933918)

[**Behavior** 4](#_Toc152933919)

[**Mental health** 4](#_Toc152933920)

[**Cognition** 4](#_Toc152933921)

[**Structural neuroimaging measures** 5](#_Toc152933922)

[**Resting-state functional connectivity** 5](#_Toc152933923)

[Method S3: Covariate 6](#_Toc152933924)

[**Age at baseline** 6](#_Toc152933925)

[**Gender** 6](#_Toc152933926)

[**Race/ethnicity** 6](#_Toc152933927)

[**Birth context score** 6](#_Toc152933928)

[**Family context score** 7](#_Toc152933929)

[**Society context score** 8](#_Toc152933930)

[Method S4: Polygenic Score (PGS) Derivation 9](#_Toc152933931)

[Method S5: Context score calculation 10](#_Toc152933932)

[Method S6: Effect size in moderation analyses 10](#_Toc152933933)

[Supplementary Figure S1: Inclusion criteria for each series of analyses in a study of prenatal substance exposure and child health from the Adolescent Brain Cognitive Development (ABCD) cohort 11](#_Toc152933934)

[Supplementary Figure S2: Dose responses of the estimated PSE-alcohol/caffeine/marijuana total consumption during pregnancy associated with child mental health and cognitive functioning, adjusting for fixed and random effects at baseline and 2-year follow-up 12](#_Toc152933935)

[References 15](#_Toc152933936)

## Method S1: Prenatal substance exposures

**ABCD Baseline Substance Use Questions During Pregnancy**

Before becoming aware of the pregnancy:

- Did you/the biological mother use tobacco?
- How many times per day did you smoke?
- Did you/the biological mother consume alcohol?
- What was the maximum number of alcoholic drinks you had in one sitting?
- How many alcoholic drinks did you typically have per week?
- Did you/the biological mother use marijuana?
- How many times per day did you use marijuana?

After becoming aware of the pregnancy:

- Did you/the biological mother use tobacco?
- How many times per day did you smoke?
- Did you/the biological mother consume alcohol?
- What was the maximum number of alcoholic drinks you had in one sitting?
- How many alcoholic drinks did you typically have per week?
- Did you/the biological mother use marijuana?
- How many times per day did you use marijuana?

Did you/the biological mother consume any caffeine during pregnancy from conception until delivery? (1 = at least once a day; 2 = less than once a day but more than once a week; 3 = less than once a week; 0 = No)

During pregnancy:

How much caffeine/day?

How much caffeine/week?

How much caffeine/month?

**Prenatal polysubstance exposure**

Polysubstance exposure during pregnancy is defined as the simultaneous exposure to more than one substance.

**Total prenatal substance consumption**

To examine the dose-dependent associations of PSEs on persistent/late-onset associations, we calculated the estimates of the total amount of caffeine consumption, number of alcoholic drinks, number of smoking times, and marijuana use throughout pregnancy. We have the following notations:

| Variable | Description |
| --- | --- |
| a | average caffeine consumption per day |
| b | average caffeine consumption per week |
| c | average caffeine consumption per month |
| d | caffeine intake frequency: daily/weekly/monthly |
| e | average number of drinks consumed per week before pregnancy knowledge |
| f | the week when the mother learned of pregnancy |
| g | average number of drinks consumed per week following pregnancy knowledge |
| h | average number of smoking times per day before pregnancy knowledge |
| i | average number of smoking times per day after pregnancy knowledge |
| j | average number of marijuana use times per day before pregnancy knowledge |
| k | average number of marijuana use times per day after pregnancy knowledge |
| l | gestational week of birth |

Two weeks were adjusted by subtracting the reported week of pregnancy knowledge to account for the conception date.

$$Total consumption estimate for daily caffeine drinker = 7\times a\left( l - 2 \right)$$

$$Total consumption estimate for \mathrm{weekly} caffeine drinker =b\left( l - 2 \right)$$

$$Total consumption estimate for \mathrm{monthly} caffeine drinker = \frac{12}{52}\times c\left( l - 2 \right)$$

$$Estimate of total alcoholic drinks =ef + g(l - f - 2)$$

$$Estimate of smoking times=hf + i(l - f - 2)$$

$$Estimate of total marijuana use times = jf + k(l - f - 2)$$

**Prenatal substance use patterns classification**

To investigate the effects of common PSE-alcohol patterns, maternal drinking was categorized as abstinent (< 1 standard drink per occasion throughout pregnancy), light (1-2 drinks per occasion, less than 7 drinks per week), moderate (3-4 drinks per occasion, less than 7 drinks per week), heavy (less than 5 drinks per occasion, 7 or more drinks per week), or binge drinking (5 or more drinks per occasion) before and after pregnancy knowledge, following the established classification(1).

Five PSE-alcohol patterns were identified:

1. Light reducers (light drinkers before pregnancy knowledge, abstinent after learning about pregnancy).
2. Heavier reducers (moderate, heavy, and binge drinkers before pregnancy knowledge, followed by abstinent or light drinking after learning about pregnancy).
3. Stable light users throughout pregnancy.
4. Stable heavier users throughout pregnancy.
5. Increasers (increased their alcohol consumption after learning about pregnancy).

In examining the effects PSE-tobacco patterns, maternal smoking was classified in terms of pack-years(2), including never smokers (0.0 pack-years), light smokers (0.1-20.0 pack-years), moderate smokers (20.1-40.0 pack-years), and heavy smokers (more than 40 pack-years) before and after pregnancy knowledge(3).

Five PSE-tobacco patterns were identified:

1. Light reducers (light smokers before knowing, quit smoking after knowing of pregnancy);
2. Heavier reducers (moderate, heavy smokers before knowing, quit smoking or light smokers after knowing);
3. Stable light users throughout pregnancy;
4. Stable heavier users throughout pregnancy;
5. Increasers (smoke more after knowledge of pregnancy).

## Method S2: Health outcomes

### **Behavior**

*Sleep Function.* Sleep Disturbance Scale for Children (4) is a 27 item Likert-type rating scale evaluating the sleep disturbances administered to a parent. The total score was calculated by summing the 6 subscales of sleep experiences and disorders.

*Impulsivity****.*** Impulsivity was graded using the Urgency, Premeditation, Perseverance, Sensation Seeking, Positive Urgency, Impulsive Behavior (UPPS-P) Scale for Children (5).

*Behavioral inhibition and behavioral activation.* Inhibition and reward seeking was examined using the Behavioral Inhibition/Behavioral Approach System (BIS/BAS) Scales(6).

### **Mental health**

*Dimensional Psychopathology/Adaptive Function.* Dimensional psychopathology was examined in children using the parent-reported Achenbach Child Behavior Check List (CBCL) using the eight empirically based syndrome scales and Achenbach Brief Problem Monitor (BPM-T)(7).

*Past/present mental disorders.* Mental disorders (i.e., diagnoses and/or symptoms, past and/or present) were determined according to parent-reported responses on the Kiddie-Structured Assessment for Affective Disorders and Schizophrenia (K-SADS) (8).

*Psychotic-like experiences (PLEs).* Psychotic-like experiences (PLEs) during childhood is measures on the Prodromal Questionnaire(9). Higher total score means more PLEs.

### **Cognition**

*Cognition****.*** NIH Toolbox Battery includes the Dimensional Change Card Sort, Flanker Inhibitory Control and Attention, List Sorting Working Memory, Oral Reading Recognition, Pattern Comparison Processing Speed, Picture Sequence Memory, and Picture Vocabulary tests and composite cognitions include total, crystallized, and fluid cognition(10). Rey Auditory Verbal Learning Test measured learning and memory recall(11). Risk-taking was examined by single-item Cash Choice Task (12).

### **Structural neuroimaging measures**

Neuroimaging measures were evaluated on 3T scanners across the ABCD study sites(13). Participants completed MRI scans of 3D T1-weighted images with correction. Cortical surface reconstruction was processed by FreeSurfer, version 5.3.0, using a standardized ABCD pipeline. In the present study, we focused on cortical volumes, surface areas and thicknesses of 68 brain regions from the Desikan-Killany atlas for analysis.

### **Resting-state functional connectivity**

The Gordon functional atlas was utilized to categorize cortical surface regions into 12 large networks, including auditory, cingulo-opercular, cingulo-parietal, default-mode, dorsal-attention, fronto-parietal, retrosplenial-temporal, salience, sensorimotor-hand, sensorimotor-mouth, ventral-attention, and visual networks(14). To determine the strength of resting-state functional connectivity (rsFC), the Fisher r-to-z transformed indices of the average correlation values were computed between pairs of regions within each large network (N=12), between these 12 networks (N=66), and between the large networks and 19 subcortical regions (N=228).

## Method S3: Covariate

### **Age at baseline**

Child age were self-reported and converted to years.

### **Gender**

Child sex is categorized as female or male at birth.

### **Race/ethnicity**

We formed five groups for the most prevalent categories of race/ethnicity from Parent Demographics Survey results (i.e., White, Black, Asian, Hispanic, and with remaining being assigned to Other).

### **Birth context score**

*Prenatal substance use.* Parents/caregivers answered whether mothers used other substances (i.e., Cocaine/Crack, Heroin/Morphine, Oxycontin) during pregnancy.

*Vitamins use.* Parents/caregivers answered whether mothers used vitamins during pregnancy.

*Planned pregnancy.* Parents/caregivers reported retrospectively whether or not they planned pregnancy.

*Duration of breastfeeding.* Parents/caregivers retrospectively answered how many months their child was breastfed.

*Prematurity.* Parents/caregivers retrospectively answered whether their child was born prematurely.

*Obstetric complications.* Parents/caregivers retrospectively reported whether any pregnancy-related complications present.

At least 1 of the following complications:

1. Severe nausea and vomiting extending past the 6th month or accompanied by weight loss
2. Heavy bleeding requiring bed rest or special treatment
3. Pre-eclampsia, eclampsia, or toxemia
4. Severe gall bladder attack
5. Persistent proteinuria
6. Rubella (German measles) during first 3 months of pregnancy
7. Severe anemia
8. Urinary tract infections
9. Pregnancy-related diabetes
10. Pregnancy-related high blood pressure
11. Problems with the placenta
12. An accident or injury requiring medical care
13. Any other conditions requiring medical care.

*Birth complications.* Parents/caregivers retrospectively reported whether any difficulties or challenges occurred during the process of childbirth.

At least 1 of the following conditions:

1. Blue at birth
2. Slow heart beat
3. Not breathe at first
4. Convulsions
5. Jaundice needing treatment
6. Required oxygen
7. Required blood transfusion
8. Rh incompatibility

### **Family context score**

*Parental age at birth.* Parents/caregivers retrospectively reported the child’s biological mother’s age and father's age at the time of the child’s birth.

*Parental partnership.* Parents/caregivers whether they having a partner who shares childcare.

*Parental education.* Parents/caregivers reported the highest education level and the variable was recoded into five distinct categories (i.e., Less than Bachelor's degree, Bachelor's degree, Master’s degree, Professional School degree and Doctoral degree).

*Family income.* Parents/caregivers reported their total combined family income for the past year. We recoded the variable into four categories based on the results (i.e., <$50,000, $50,000–100,000, >$100,000 and with remaining being assigned to Other).

*Parental psychology.* Parents/caregivers self-reported their own adaptive functioning.

*Parental monitoring.* The score of parental monitoring and supervision was calculated as the average of the five questions from the ABCD Parental Monitoring Survey(15).

*Family Conflict.* Family conflict was derived from the Parental Monitoring Questionnaire Family Environment(16).

### **Society context score**

*Drug availability.* Parents/caregivers reported the ease of access and exposure to drug (alcohol, nicotine, marijuana, “other” drugs) in the community.

*Child opportunity score.* Nationally-normed Child Opportunity Scores (from 1 to 100) for the education, health and environment, social and economic and overall domain.

*Lead risk.* Estimated lead risk in census tract at current address (1-10 scale).

*Air pollution.* Annual average of PM 2.5 in 2016 at current residential address at 1x1km2.

*Noise.* Average Annual Daily Traffic Counts at current address was categorized into three groups, including <1,000, 1,000 –8,000 and >8,000.

*Urbanicity*. Census Tract Urban Classification at current address (i.e., 1 = Rural; 2 = Urban Clusters; 3 = Urbanized Area).

*Neighborhood safety from crime.* Youth reported the safety from crime of neighborhood which is the area within about a 20-minute walk (or about a mile) from home.

*School environment.* Youth completed The School Risk and Protective Factors questionnaire and the school environment score was derived(17).

## Method S4: Polygenic Score (PGS) Derivation

For the calculation of polygenic risk score (PRS) for attention deficit/hyperactivity disorder (ADHD)(18), major depression (MDD)(19), schizophrenia (SCZ)(20), educational attainment (EDU)(21), alcohol dependence (ALCDEP)(22) and cannabis use disorder (CUD)(23), we used summary statistics generated from the most well-powered discovery genome-wide association studies (GWASs) from the Psychiatric Genomics Consortium as the discovery sample.

Genotyped calls were aligned with GRCH37(hg19). SNPs with minor allele frequency (MAF) below 1% or imputation information score (INFO) below 0.8 were excluded from the PRS analyses. SNPs with call rates ≥ 0.95 and MAF ≥ 1% were retained. Individuals with high missingness rates (> 5%) and autosomal heterozygosity deviations (FHET) not within ± 3 SD were excluded; SNPs were further filtered for call rates ≥ 0.98 and Hardy-Weinberg p values > 1E-6 (founders only) after sample quality control. Sex checks were performed to remove mismatches between biological and reported sex for follow-up analyses(24).

The final PCA of ancestral information was performed after selection and the first 15 PCs were projected from the founders to other relatives. Initially 516,598 SNPs were shared by the discovery sample and the ABCD dataset, and 350,622 SNPS were generated after clumping. Dosage data were converted to hard-call genotypes using the genetic analyses tool Plink (v1.90), and only SNPs with imputation r2 scores ≥ 0.3 were transferred to PRS(25). We used Plink to calculate PRS under P-value thresholds at 0.001, 0.05, 0.10, 0.20, 0.30, 0.40, 0.50 (26) and obtained the mean of all P-value thresholds of the relevant PRS.

In analyses, we included all fixed-effect covariates and the first 15 principal components (PCs) when running linear mixed effect models for each outcome of interest and all models nested data by family membership within site.

## Method S5: Context score calculation

After selecting the covariates, we first classified them into two groups according to their attributes: risky and protective. Then the raw data of the variables were linearly transformed using min-max normalization to control the range of each environmental/genetic factor between 0 and 1, with a higher score indicating greater risk of the factor. Then, the transformed environmental/genetic factors were added together by context to obtain the initial birth/family/society/genetic context score. Last, the birth/family/society/genetic context score were mean centered to remove the unnecessary multicollinearity between the interaction terms and their component variables in moderation analyses.

## Method S6: Effect size in moderation analyses

The moderating effect implies that the magnitude of the association between predictor *X* and outcome variable *Y* depends on the value of a third variable, *Z*, the moderator. The effect of *X* on *Y* varies with the value of *Z*. We used a new effect size in our moderation analysis to quantify the magnitude of the moderating effect, reflecting the impact of *Z* on the relationship between *X* and *Y* (27).

The combined form of the developed 2MMR model is

$Y_{i}= \gamma_{00}+ \gamma_{01}Z_{i}+\left( \gamma_{10}+\gamma_{11}Z_{i}+u_{i1} \right)X_{i}+\delta_{i}$ ,

where ${\gamma_{00}, \gamma_{01}, \gamma}_{10}$, $\gamma_{11}$ are regression coefficients(28).

Thus, the new effect size is defined based on the explained variance of *Y* via *X*, that is

${VR}^{2}=\frac{variance explained by the path of XZ\mathrm{to}Y}{explained variance of Y\mathrm{via}X}$ $=\frac{Var\left( \gamma_{11}X_{i}Z_{i} \right)}{Var\left[ \left( \gamma_{10}+\gamma_{11}Z_{i} \right)X_{i} \right]}$ .

*VR*^2^ is the ratio of the variance explained by the effect of *XZ* on *Y* to the explained variance of *Y* via *X*. We also applied a transformation to minimize *Var*(*xz*) to avoid all variance ratios beyond the range of 0 to 1(27).


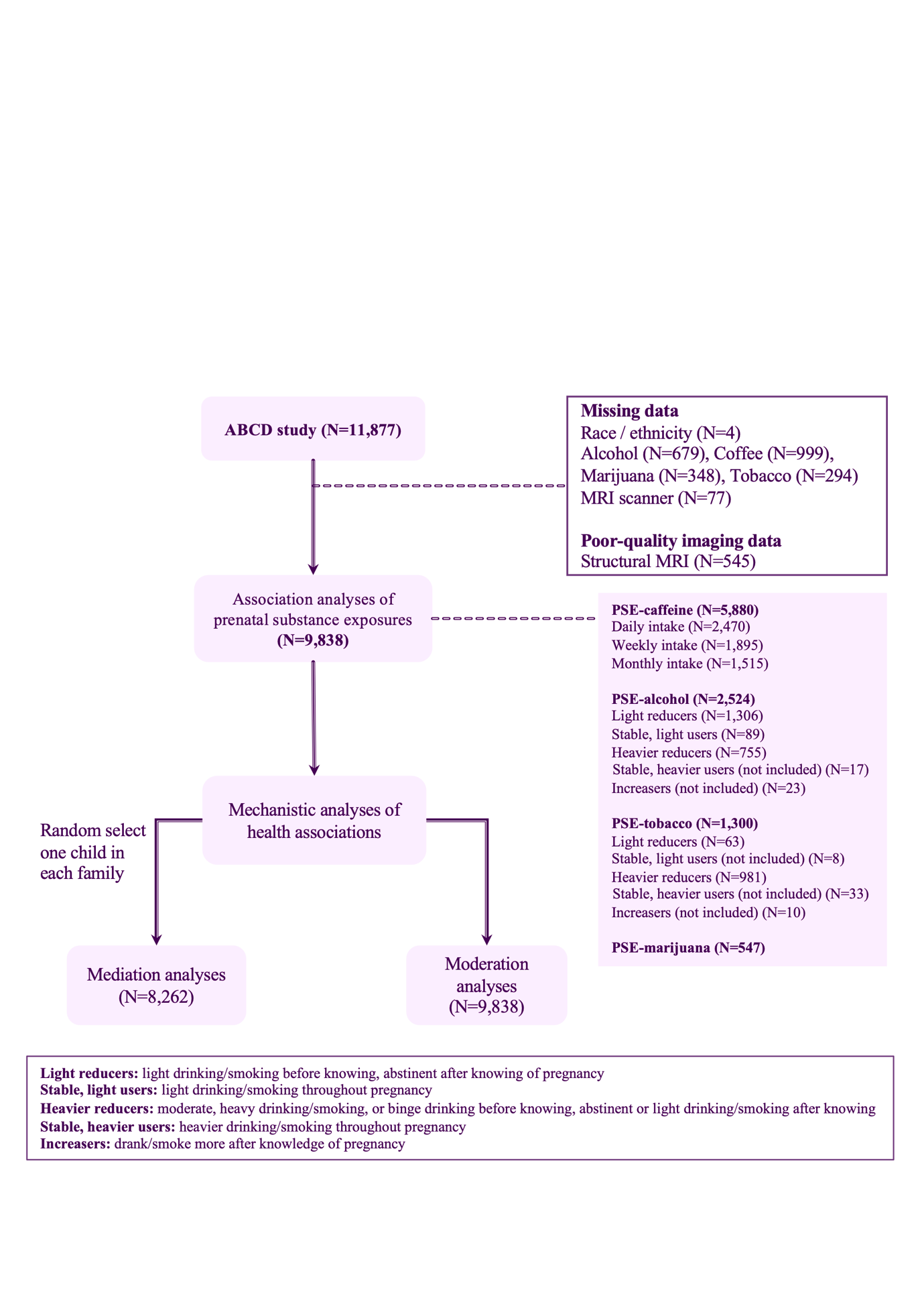


## Supplementary Figure S1: Inclusion criteria for each series of analyses in a study of prenatal substance exposure and child health from the Adolescent Brain Cognitive Development (ABCD) cohort

## Supplementary Figure S2: Dose responses of the estimated PSE-alcohol/caffeine/marijuana total consumption during pregnancy associated with child mental health and cognitive functioning, adjusting for fixed and random effects at baseline and 2-year follow-up


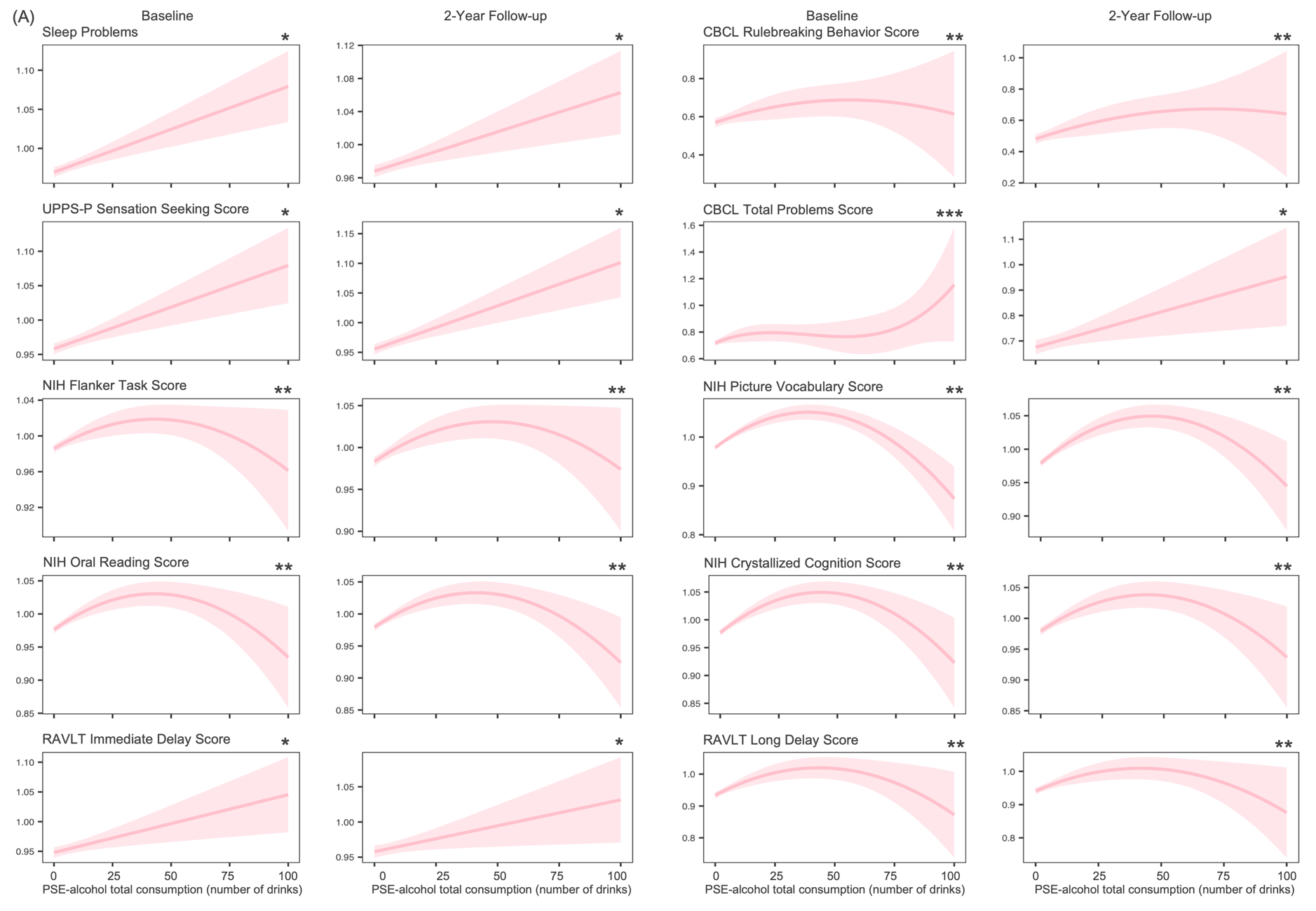


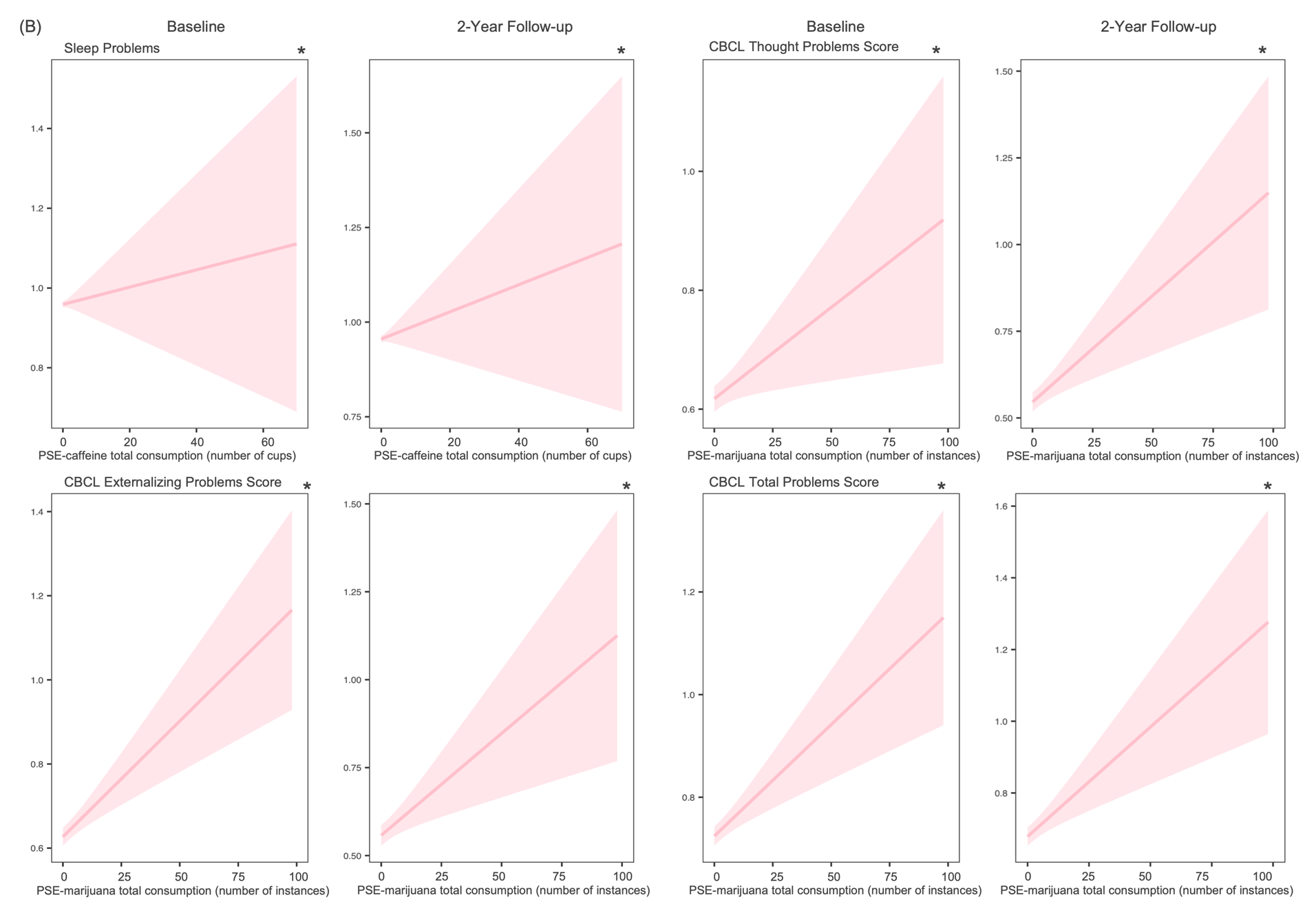


(A) PSE-alcohol consumption was associated with higher sensation seeking, more sleep problems and CBCL-total problems in children. PSE-alcohol consumption also demonstrated nonlinear dose-response associations in both CBCL-total problems and cognitive functions. Most pregnant women (92.4%) who drank alcohol had less than 75 drinks during pregnancy, showing linear dose-responses. For the 93 women (7.6%) with over 75 drinks, higher consumption was associated with increased CBCL-total problems and lower cognitive functions, such as performance in NIH Flanker Task, Picture Vocabulary, Oral Reading and Crystallized Cognition and RAVLT Long Delay. More data is needed to confirm the effect of high alcohol consumption during pregnancy (i.e., above 75 drinks) on children's cognitive development. (B) More PSE-caffeine consumption was associated with more child sleep problems. More frequent PSE-marijuana was associated with more thought, externalizing, and total problems. Significant linear associations were marked with *, significant quadratic associations with **, and significant cubic associations with ***.

## References

1. C M O’Leary, *et al.*, A new method of prenatal alcohol classification accounting for dose, pattern and timing of exposure: improving our ability to examine fetal effects from low to moderate alcohol. *J Epidemiol Community Health* **64**, 956 (2010).

2. J. F. Price, *et al.*, Relationship between smoking and cardiovascular risk factors in the development of peripheral arterial disease and coronary artery disease; Edinburgh Artery Study: Edinburgh Artery Study. *European Heart Journal* **20**, 344–353 (1999).

3. Y.-H. Lee, *et al.*, Cumulative smoking exposure, duration of smoking cessation, and peripheral arterial disease in middle-aged and older Korean men. *BMC Public Health* **11**, 94 (2011).

4. O. BRUNI, *et al.*, The Sleep Disturbance Scale for Children (SDSC) Construct ion and validation of an instrument to evaluate sleep disturbances in childhood and adolescence. *Journal of Sleep Research* **5**, 251–261 (1996).

5. S. P. Whiteside, D. R. Lynam, J. D. Miller, S. K. Reynolds, Validation of the UPPS impulsive behaviour scale: a four-factor model of impulsivity. *European Journal of Personality* **19**, 559–574 (2005).

6. D. Pagliaccio, *et al.*, Revising the BIS/BAS Scale to study development: Measurement invariance and normative effects of age and sex from childhood through adulthood. *Psychological Assessment* **28**, 429–442 (2016).

7. T. M. Achenbach, “Achenbach System of Empirically Based Assessment (ASEBA)” in *Encyclopedia of Clinical Neuropsychology*, J. Kreutzer, J. DeLuca, B. Caplan, Eds. (Springer International Publishing, 2018), pp. 1–7.

8. L. Townsend, *et al.*, Development of Three Web-Based Computerized Versions of the Kiddie Schedule for Affective Disorders and Schizophrenia Child Psychiatric Diagnostic Interview: Preliminary Validity Data. *Journal of the American Academy of Child & Adolescent Psychiatry* **59**, 309–325 (2020).

9. N. R. Karcher, *et al.*, Assessment of the Prodromal Questionnaire–Brief Child Version for Measurement of Self-reported Psychoticlike Experiences in Childhood. *JAMA Psychiatry* **75**, 853–861 (2018).

10. R. C. Gershon, *et al.*, NIH Toolbox for Assessment of Neurological and Behavioral Function. *Neurology* **80**, S2 (2013).

11. E. Strauss, E. M. S. Sherman, O. Spreen, *A compendium of neuropsychological tests: Administration, norms, and commentary, 3rd ed.* (Oxford University Press, 2006).

12. E. Wulfert, J. A. Block, E. S. Ana, M. L. Rodriguez, M. Colsman, Delay of gratification: Impulsive choices and problem behaviors in early and late adolescence. *Journal of Personality* **70**, 533–552 (2002).

13. B. J. Casey, *et al.*, The Adolescent Brain Cognitive Development (ABCD) study: Imaging acquisition across 21 sites. *Dev Cogn Neurosci* **32**, 43–54 (2018).

14. E. M. Gordon, *et al.*, Generation and Evaluation of a Cortical Area Parcellation from Resting-State Correlations. *Cerebral Cortex* **26**, 288–303 (2016).

15. H. C. Karoly, T. Callahan, S. J. Schmiege, S. W. Feldstein Ewing, Evaluating the Hispanic Paradox in the Context of Adolescent Risky Sexual Behavior: The Role of Parent Monitoring. *Journal of Pediatric Psychology* **41**, 429–440 (2016).

16. B. S. Moos, R. H. Moos, *Family Environment Scale Manual: Development, Applications, Research*, 3rd ed (Consulting Psychologists Press, 1994).

17. M. W. Arthur, *et al.*, Measuring risk and protection in communities using the Communities That Care Youth Survey. *Evaluation and Program Planning* **30**, 197–211 (2007).

18. D. Demontis, *et al.*, Discovery of the first genome-wide significant risk loci for attention deficit/hyperactivity disorder. *Nat Genet* **51**, 63–75 (2019).

19. N. R. Wray, *et al.*, Genome-wide association analyses identify 44 risk variants and refine the genetic architecture of major depression. *Nat Genet* **50**, 668–681 (2018).

20. V. Trubetskoy, *et al.*, Mapping genomic loci implicates genes and synaptic biology in schizophrenia. *Nature* **604**, 502–508 (2022).

21. J. J. Lee, *et al.*, Gene discovery and polygenic prediction from a genome-wide association study of educational attainment in 1.1 million individuals. *Nat Genet* **50**, 1112–1121 (2018).

22. R. K. Walters, *et al.*, Transancestral GWAS of alcohol dependence reveals common genetic underpinnings with psychiatric disorders. *Nat Neurosci* **21**, 1656–1669 (2018).

23. E. C. Johnson, *et al.*, A large-scale genome-wide association study meta-analysis of cannabis use disorder. *The Lancet Psychiatry* **7**, 1032–1045 (2020).

24. M. Lam, *et al.*, RICOPILI: Rapid Imputation for COnsortias PIpeLIne. *Bioinformatics* **36**, 930–933 (2020).

25. Y. Li, C. J. Willer, J. Ding, P. Scheet, G. R. Abecasis, MaCH: using sequence and genotype data to estimate haplotypes and unobserved genotypes. *Genetic Epidemiology* **34**, 816–834 (2010).

26. S. W. Choi, T. S.-H. Mak, P. F. O’Reilly, Tutorial: a guide to performing polygenic risk score analyses. *Nat Protoc* **15**, 2759–2772 (2020).

27. H. Liu, K.-H. Yuan, New measures of effect size in moderation analysis. *Psychological Methods* **26**, 680–700 (2021).

28. K.-H. Yuan, Y. Cheng, S. Maxwell, Moderation Analysis Using a Two-Level Regression Model. *Psychometrika* **79**, 701–732 (2014).
